# Efficacy of eHealth Interventions for Adults with Diabetes: A Systematic Review and Meta-Analysis

**DOI:** 10.3390/ijerph18178982

**Published:** 2021-08-26

**Authors:** Giulia Bassi, Elisa Mancinelli, Gaia Dell’Arciprete, Silvia Rizzi, Silvia Gabrielli, Silvia Salcuni

**Affiliations:** 1 Department of Developmental and Socialization Psychology, University of Padova, Via Venezia 8, 35131 Padova, Italy; giulia.bassi@phd.unipd.it (G.B.); dellarcigaia@libero.it (G.D.); silvia.salcuni@unipd.it (S.S.); 2 Digital Health Lab, Centre for Digital Health and Wellbeing, Fondazione Bruno Kessler, Trento, Via Sommarive 18, 38123 Povo, Italy; srizzi@fbk.eu (S.R.); sgabrielli@fbk.eu (S.G.)

**Keywords:** diabetes mellitus, eHealth, psychosocial factors, HbA1c, meta-analysis

## Abstract

The aim is to provide meta-analytical evidence on eHealth interventions’ efficacy in supporting the psychosocial and physical well-being of adults with type 1 or type 2 Diabetes Mellitus (DM), and to investigate differences in interventions primarily targeted at providing glycemic control vs. psychosocial support. A PRISMA-guided systematic search was conducted. Randomized Controlled Trials (RCTs) regarding eHealth interventions for adults (18–65 years) with DM were included. Data were pooled using Standard Mean Difference (SMD); sub-group analysis and meta-regressions were performed when appropriate. Outcomes were Hemoglobin A1c (HbA1c), diabetes distress, quality of life, anxiety, stress, and depression. Intervention acceptability was assessed performing the Odds Ratio (OR) of drop-out rates. Thirteen RCTs comprising 1315 participants were included (52.09% females; M_age_ = 46.18, SD = 9.98). Analyses showed intervention efficacy on HbA1c (SMD = −0.40; 95% CI = −0.70, −0.12; k = 13) and depressive symptoms (SMD = −0.18; 95% CI = −0.33, −0.02; k = 6) at RCTs endpoint and were well accepted (OR = 1.43; 95% CI = 0.72, 2.81; k = 10). However, efficacy on HbA1c was not maintained at follow-up (SMD = −0.13; 95% CI = −0.31, 0.05; k = 6). eHealth interventions providing medical support were acceptable and effective in fostering glycemic control and decreasing depressive symptoms in the short-term only. Digital solutions should be developed on multiple levels to fully support the psychophysical well-being of people with DM.

## 1. Introduction

Diabetes Mellitus (DM) is a chronic metabolic disease, which has a significant impact on issues related to clinical, social, and economic factors, as well as on people’s quality of life, thereby leading to increased morbidity and mortality [1,2]. In particular, Type 1 DM (T1DM), once referred to as juvenile diabetes or insulin-dependent diabetes mellitus, is a chronic autoimmune condition in which the pancreas is not able to produce enough insulin due to the loss of beta cells [2]. On the other hand, the most common form is Type 2 DM (T2DM), once referred to as adult-onset diabetes or non-insulin-dependent diabetes mellitus, which occurs when the body becomes resistant to insulin, namely when cells fail to respond to insulin properly [2]. The World Health Organization (WHO) estimated that the global prevalence of DM was at 8.5% among adults of 18 years and above, and 422 million adults were living with DM, compared to 108 million in 1980 [3]. The WHO further reports that deaths from diabetes increased by 70% worldwide between 2000 and 2019, with mortality rates being greater among males as compared to females, cross-culturally and independently of age [4].

Most studies have found evidence that diabetes and its complications can be prevented by introducing healthier lifestyle changes [5]. Psychosocial factors have an impact on quality of life and often influence chronic disease outcomes; for this reason, the healthy coping construct was included, in line with the awareness that psychological distress affects the general health of people with DM and, thus, affects their motivation to keep their chronic disease under control. In this regard, clinical recommendations for effective self-care behaviors are deemed particularly challenging to maintain, and the associated barriers (i.e., non-adherence and treatment non-compliance) appear to be difficult to untangle. Therefore, coping strategies turn out to be pivotal resources for people with DM to better manage their disease. As such, motivation represents a core component to acquire these coping skills. Diabetes educators play an important role in the identification of persons’ motivation to support their behavior change by helping them to set behavioral goals and guiding them in confronting their barriers [6].

In this context, digital solutions can further support people with diabetes by encouraging and motivating them to better manage their health. In recent decades, the high availability of digital solutions for DM, such as smartphone-based applications, has been an important resource to provide highly accessible and low-cost personalized care; consequently, it has improved the monitoring and communication of various biometric information relevant to disease management, and fostered involvement of patients in their self-care [7,8,9,10]. Most of the digital solutions developed have focused on the monitoring of physical factors associated with Hemoglobin A1c (HbA1c), diet, physical activity, and adherence to the prescribed medication. For instance, a systematic review of Randomized Controlled Trials (RCTs) investigated computerized educational programs’ efficacy in enhancing the diet and metabolic measures of people with DM [11], while four recent meta-analyses evaluated telemedicine interventions aimed at supporting specific aspects of diabetes-management [12,13,14,15]. More specifically, one study has evaluated the efficacy of telemedicine in improving glycemic control among adults, children, and adolescents with T1DM [15]; another study has analyzed the efficacy of smartphone-based interventions for people with T2DM, reporting beneficial effects on self-efficacy, self-care activities, health-related quality of life and glycemic control [12]; the other two studies have both assessed the efficacy of eHealth interventions on glycemic control [13,14], although Ferigerlovà et al. [14] only focused on people with T1DM, while Bonoto et al. [13] considered both types of DM and further evaluated health-related quality of life as a secondary outcome. In this regard, studies have also found that high levels of anxiety, depression, and stress are associated with reduced quality of life leading to poor disease outcomes [16,17,18,19]. For instance, the literature suggests that people with DM are two to three times more likely to develop depression [20]. Concurrently, co-morbid depression in older patients with T2DM carries an increased risk of developing cognitive impairment, which can exacerbate the vicious cycle of self-criticism with consequences on self-care [21]. Then, depression predicts, together with diabetes distress, medication adherence, proper dieting and physical activity [17]. In light of this evidence, one can assume bidirectionality between psychological and physical effects, meaning that they influence one another.

Although, as just mentioned, many studies have investigated the physical and medical factors involved in diabetes management, there seems to be a lack of research on the impact of psychosocial factors, such as healthy coping. Therefore, there is a need for evidence regarding the efficacy of digital solutions concurrently supporting both the psychosocial, physical and medical well-being of people with DM, to further guarantee the choice of appropriate treatment, tailored to their needs. With this in mind, this is the first meta-analysis aimed at providing evidence gathered from RCTs evaluating eHealth interventions for adults with T1DM or T2DM that account for both medical and psychosocial variables. Specifically, the primary aim was to assess the efficacy of eHealth interventions in reducing diabetes distress and distress-related symptoms—including symptoms of depression, anxiety, and stress—and improving patients’ quality of life and HbA1c levels, thereby fostering both the psychosocial well-being and glycemic control of adults with DM. The secondary aim was then to determine whether there are differences in the efficacy of eHealth interventions primarily aimed at providing psychosocial support vs. those primarily aimed at improving glycemic control.

## 2. Materials and Methods

The current meta-analysis was conducted following the Preferred Reporting Items for Systematic Reviews and Meta-Analyses (PRISMA) guidelines [22] and following the recommendations of the Cochrane handbook for systematic reviews [23]. The protocol for this meta-analysis was approved and registered on PROSPERO in March 2021 (Registration Number: CRD42021238090).

### 2.1. Eligibility Criteria

Studies that met any of the following criteria were included in the current meta-analysis: (a) RCTs comparing any eHealth intervention with any control condition (e.g., no intervention group, waiting list, treatment as usual (TAU), active conditions); (b) adults aged between 18 and 65 years, diagnosed with T1DM or T2DM. Studies were excluded if they involved women with gestational diabetes, adolescents, children, groups of adults with other medical or psychological disorders different from or comorbid to T2DM or T1DM, and adults at risk of DM or with prediabetes.

### 2.2. Primary and Secondary Outcomes

Primary outcomes were mean changes in both psychosocial (i.e., diabetes distress, depression, anxiety, stress, and quality of life) and medical measurements (i.e., HbA1c levels). Time points included in this meta-analysis are endpoints defined as the end of the eHealth intervention and follow-up (when assessed). Secondary outcomes were the results of two different sensitivity analyses: one designed to assess the efficacy of eHealth interventions primarily aimed at providing psychosocial support, and one designed to assess the efficacy of eHealth interventions primarily aimed at improving glycemic control. Acceptability of the intervention was measured by analyzing drop-out rates.

### 2.3. Search Strategy

The literature was systematically searched via Web of Science, PubMed, and CENTRAL, using the following search terms: diabetes mellitus, eHealth, Mobile Health, mHealth, Telehealth, serious games, glyc*, glucose, HbA1c, anxiety, stress, distress, depression, quality of life, adults, randomized controlled trial, experimental, clinical. A further manual search was implemented to identify any other relevant articles not found through the mentioned academic databases. The search was limited to articles written in English and published in peer-reviewed scientific journals between 2000 and 2021. The resulting abstracts were screened independently by two authors (GB and SR) through a double-blind process; potentially eligible articles were then read in full text by two authors (GB and GD), using again the double-blind process. Any disagreement concerning both abstract and full-text articles was resolved by discussion or by consulting a third author (EM) until consensus was achieved.

### 2.4. Data Extraction

Data extraction was carried out independently by two authors (GB and GD), gathering the following data from the full texts of the included studies: first author’s name, year of publication, geographical location of the study, population characteristics (i.e., N, age, gender, type of diabetes and its duration), type and length of eHealth intervention (e.g., smartphone-based application, Internet-based system, mobile-health intervention), type of control condition (i.e., waitlist/no treatment, TAU, active), type of analyses performed in the studies (intention-to-treat (ITT) or per-protocol), drop-out rates, study’s primary and secondary outcomes, mean and standard deviation of every outcome measurement at baseline, endpoint and follow-up (when included) and, if reported, effect size with 95% Confidence Intervals (CIs) for each outcome in both study arms.

### 2.5. Quality Assessment

The Risk of Bias (RoB) of the included RCTs was independently assessed by two authors (GB and EM), using Cochrane’s Risk of Bias tool version 2 (RoB 2.0). Individual studies were judged on the following domains: randomization process, deviations from the intended interventions (effect of *assignment* to intervention; effect of *adhering* to intervention), missing outcome data, measurement of outcome data and selection of reported results. Each domain’s risk was rated as either “low”, “some concern” or “high”, depending on whether or not the requirements were adequately fulfilled. Discrepancies between evaluations were resolved by discussion or by consulting a third author (GD). Every domain’s assessment merges in a comprehensive domain, named overall judgment, included in the RoB 2.0 to provide a summary of the whole study’s quality assessment.

### 2.6. Data Analysis

Analyses were performed using Review Manager Version 5 [24] and Comprehensive Meta-Analysis [25]. Outcomes at the endpoint and follow-up were meta-analyzed when at least three studies provided the necessary data. SMD with 95% CI was calculated. Heterogeneity was assessed using I^2^, with values greater than 50% indicating heterogeneity. As such, outcomes were assessed using a random effect model when I^2^ > 50%, while the fixed effect model was used when studies showed I^2^ < 50%. Odds Ratio (OR) with 95% CI was calculated to assess intervention acceptability based on drop-out rates. Drop-out rates are usually considered as a measure of treatment acceptability [26,27].

Publication bias was assessed by visually inspecting the funnel plot and through the Egger’s regression test [28,29]; when publication bias emerged, the trim and fill procedures and the fail-safe number [30] were calculated, thereby evaluating whether results remained unchanged when accounting for publication bias. Sub-group analyses were performed based on T1DM vs. T2DM, control conditions (i.e., waiting list/no treatment, TAU, active), eHealth intervention format (i.e., smartphone-based application, internet-based telemedicine system, mobile-health intervention), and type of analysis (i.e., ITT vs. per-protocol). Meta-regression was performed when at least ten studies provided moderator data; moderators were samples’ age, gender, diabetes duration (assessed in years), and intervention duration (assessed in weeks).

## 3. Results

### 3.1. Search Result

**Figure 1:**
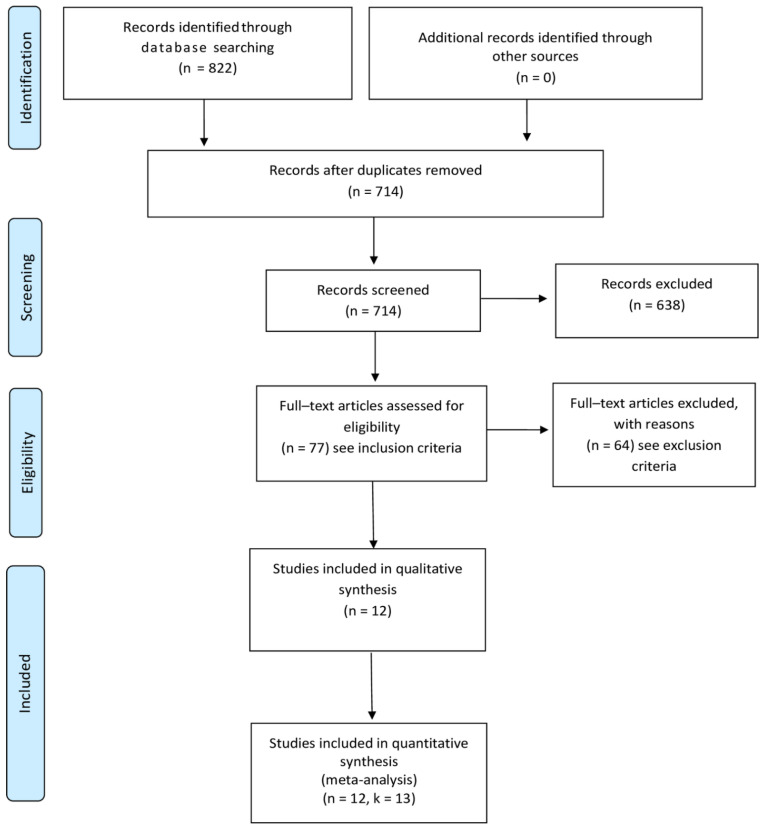
Preferred Reporting Items for Systematic Reviews and Meta-Analyses (PRISMA Statement) [22].

The search process is shown in Figure 1 and the PRISMA checklist is reported in the Supplementary Material Reporting Checklist file (Figure S1). The initial search yielded 822 studies. After duplicates were removed, the titles and abstracts of 714 studies were screened resulting in 77 studies considered for full-text screening. Following the exclusion of 64 studies (see, Supplementary Material Table S1 for the full list of excluded studies and reasons for exclusion), 12 studies reporting data on k = 13 RCTs were finally included and meta-analyzed. In this regard, as shown in Table 1, among the included 12 studies, Trief et al. [31,32] separately assessed the efficacy of two experimental interventions considering different samples. As such, the study of Trief et al. [31,32] has been considered as two separate RCTs, ultimately resulting in a total of k = 13 RCTs included.

### 3.2. Studies’ Characteristics

Included RCTs’ characteristics are reported in Table 1, which shows data collected between 2006 and 2020 [31,32,33,34,35,36,37,38,39,40,41,42,43]. A total of N = 1315 adults were considered in the current meta-analysis, of which N = 725 received the experimental intervention and N = 590 were under the control condition. Overall, 52.09% of adults were females, although one RCT [38] did not report its participants’ gender distribution (the percentage is evaluated on the remaining 12). Adults’ mean age was 46.18 years (SD = 9.98), and their mean diabetes duration was 13.60 years (SD = 8.28). Only one study [41] did not provide information on the sample’s diabetes duration. Moreover, k = 6 RCTs considered participants with T1DM [33,34,35,38,40,41] and k = 7 with T2DM [31,32,36,37,39]. All studies provided data on participants’ glycemic levels (i.e., HbA1c) assessed at the intervention endpoint, and k = 6 RCTs also evaluated it at follow-up [31,33,36,40,41]. The psychosocial variables relevant for the current meta-analysis and considered in the included RCTs were: quality of life referred to the participants’ diabetes management (k = 7) [32,33,34,35,38,40,42], diabetes distress (k = 4) [31,39,42] and depressive symptoms (k = 6) [31,32,36,37,42,43]. None of the included studies provided data on participants’ quality of life, diabetes distress, or depressive symptoms at follow-up. No data regarding anxiety nor stress symptoms were evaluated in the identified studies.

Moreover, only in three of the included studies a preliminary pilot study was conducted to assess the intervention feasibility and acceptability [31,32,33,34].

### 3.3. eHealth Interventions’ Characteristics

eHealth interventions’ characteristics are described in Table 2. All interventions were behavioral and primarily aimed at improving glycemic control and self-management, with only four RCTs that also considered and assessed the efficacy of social support [31,32,42] and quality of life [33,34]. No study was primarily aimed at providing psychosocial support to improve the considered psychosocial variables (i.e., quality of life, diabetes distress, depressive symptoms). The delivery format of the eHealth interventions comprises Short Message Service (SMS) (k = 1) [35], phone calls (k = 3) [31,32,36], video calls (k = 1) [37] and web, smartphone or computer-based applications (k = 8) [31,34,38,39,40,41,42,43].

Control conditions were waiting list (k = 1) [35], TAU (k = 8) [33,34,36,37,38,40,41,43] and active control condition (k = 4) [32,38,39,42]. Lastly, k = 7 RCTs considered all randomized participants for analysis (ITT) [31,32,33,34,36,40,41], while k = 6 considered only the observed case (per-protocol) [35,37,38,39,42,43]. Additionally, none of the included studies relied on behavior change theories, to the exception of one study. This latter study is based on the Social Learning Theory (comprising knowledge development, goal setting, self-monitoring, and behavioral contracting) and on the Interdependence Theory only for the Couple Calls intervention [31,32].

### 3.4. Quality Assessment of Included Studies

**Figure 2:**
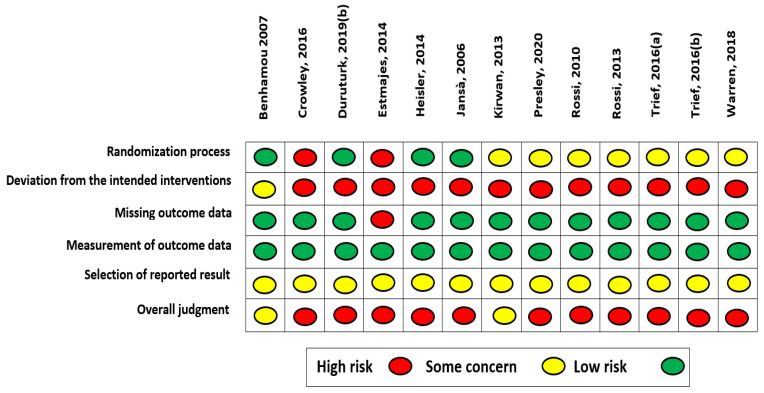
Risk of Bias color-coded.

RoB assessment of included RCTs is reported in Figure 2 (a second Figure picturing RoB results in an aggregated form per domain are reported in Supplementary Material Figure S2). Overall, studies quality yielded a high RoB (k = 11) and only k = 2 RCTs showed some concern. The primary sources of bias were the domain investigating deviation from the intended intervention (84.62% high RoB, 15.38% some concern), and the selection of reported results domain (100% some concern). For the latter, all studies were deemed as holding some concern, as most of the studies did not provide a pre-specified protocol, while those that did not provide information on the intended analyses to be performed. Other sources of bias refer to the randomization process domain (15.38% high RoB, 53.85% some concern).

### 3.5. Interventions’ Efficacy and Meta-Regression

#### 3.5.1. Hemoglobin 1c (HbA1c)

**Figure 3:**
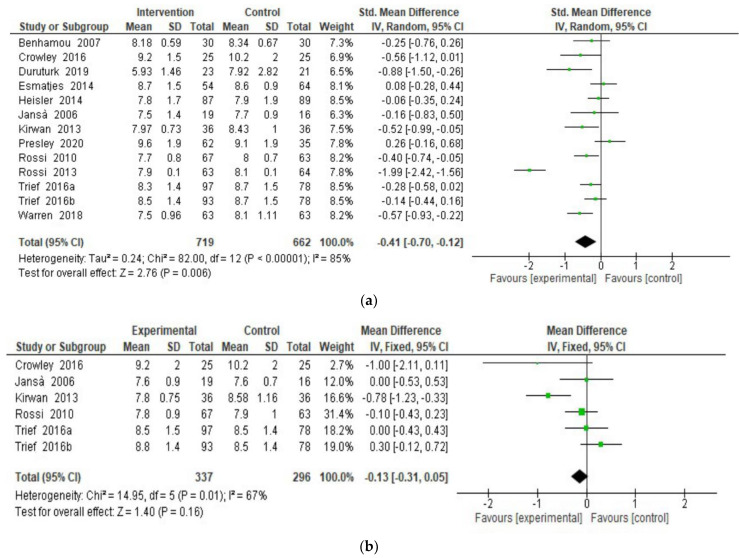
(**a**)**.** HbA1c at endpoint. (**b**). HbA1c at follow-up.

A random-effect meta-analysis was performed to assess eHealth interventions’ efficacy in improving participants’ HbA1c levels. Results showed a significant effect on HbA1c (Figure 3a) at intervention endpoint (SMD = −0.40; 95% CI = −0.70, −0.12; I^2^ = 85%; k = 13) [31,32,33,34,35,36,37,38,39,40,41,42,43], favoring participants in the intervention group, thereby showing more balanced (i.e., within the optimal range of glycemic level) glycemic levels compared to the higher glycemic levels reported by participants in the control group. Overall, interventions were well-accepted (drop-out OR = 1.43; 95% CI = 0.72, 2.81; I^2^ = 74%; k = 10), but the beneficial effect on HbA1c was not maintained at follow-up (Figure 3b; SMD = −0.13; 95% CI = −0.31, 0.05; I^2^ = 67%; k = 6) [31,32,33,36,40,41], as reported in Figure 3b.

As displayed in Table 3, subgroup analysis was then performed, considering the type of DM (i.e., T1DM vs. T2DM), control condition (i.e., waiting list/no treatment, TAU, active control group), intervention delivery format (i.e., SMS, phone calls, video calls, applications) and the type of analysis (i.e., ITT vs. per-protocol). No significant difference in the effect size was shown. In addition, as indicated in Table 3, meta-regression results showed no significant moderator (i.e., age, gender, DM duration, and intervention duration).

#### 3.5.2. Psychosocial Outcomes

**Figure 4:**
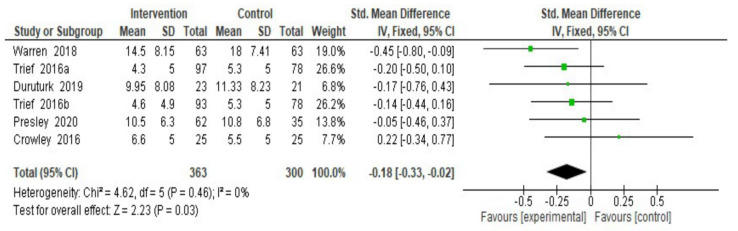
Depressive symptoms at the endpoint. Note. SD = Standard Deviation; Std. Mean Difference= Standard Mean Difference; CI = Confidence Interval; df = degrees of freedom.

eHealth interventions’ efficacy on psychosocial outcomes (i.e., depressive symptoms, quality of life, diabetes distress) was also assessed; since no heterogeneity was found for depressive symptoms, a fixed-effect meta-analysis was only performed for this outcome. Results showed a significant effect on participants’ depressive symptoms at endpoint (Figure 4; SMD = −0.18; 95% CI = −0.33, −0.02; I^2^ = 0%; k = 6) [31,32,36,37,42,43]. Data on depressive symptoms at follow-up were only provided by one RCT [36], thus the interventions’ efficacy at follow-up could not be assessed for this outcome.

**Figure 5:**
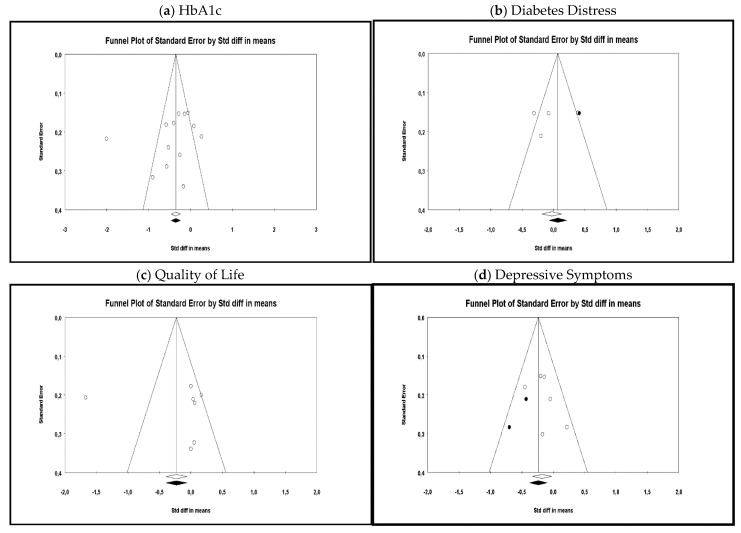
Publication bias Funnel Plots (observed and imputed). Note. Each dot represents a single RCT; Y-axis = the standard error (RCTs with lower power are shown at the bottom, those with higher power at the top of the funnel); X-axis = the SMD; an asymmetrical dots distribution suggest bias.

A random-effect meta-analysis was instead performed to assess interventions’ efficacy on participants’ quality of life (Figure 5; SMD = −0.20; 95% C I = −0.72, 0.32; I^2^ = 90%; k = 7) [31,32,39,42] and diabetes distress (SMD = −0.04; 95% CI = −0.36, 0.27; I^2^ = 74%; k = 4) [33,34,35,38,40,42,43] at interventions’ endpoint. As regards both quality of life and diabetes distress, no significant effect was found among people with DM. No data was reported to assess interventions’ efficacy at follow-up for either quality of life or diabetes distress. The figures related to both outcomes are reported in the Supplementary Materials Figures S3 and S4, respectively.

None of the sub-group analysis concerning the above-mentioned comparisons showed significant differences in the effect size for any of the considered psychosocial outcomes. Meta-regression could not be performed as less than 10 studies provided the necessary data.

#### 3.5.3. Publication Bias

Publication bias was assessed for all considered outcomes as shown in Figure 5. The Egger’s regression test was also performed, and no publication bias was found, referring to either HbA1c (β_0_ = −2.95; *p* = 0.17), quality of life (β_0_ = 1.53; *p* = 0.41) or diabetes distress (β_0_ = −3.66; *p* = 0.36). Egger’s regression test, instead, revealed the presence of significant publication bias among RCTs assessing depressive symptoms (β_0_ = −8.26; *p* = 0.02). Hence, the trim and fill procedures were performed, trimming two studies to the left (Point estimate = −1.31; 95% CI = −1.44, −1.18). The fail-safe N was equal to 54. These results suggest that the interventions’ effect on depressive symptoms was not influenced by the presence of publication bias.

## 4. Discussion

The present meta-analysis examined the efficacy of eHealth interventions in improving psychosocial outcomes and glycemic control in patients with DM, including data from 13 RCTs of 1315 adults with T1DM or T2DM. The current study further aims to determine whether there were differences in the efficacy of eHealth interventions in fostering psychosocial support (i.e., decreasing diabetes distress, stress, anxiety, depression symptoms, and increasing quality of life) and improving HbA1c levels for adults with DM. Referring specifically to the goal of investigating the efficacy of eHealth interventions aimed at providing psychosocial support, such intent could not be satisfied, as none of the included studies was designed accordingly. On the other hand, all included studies were focused on supporting glycemic control by favoring diabetes management.

Overall, the results demonstrated the efficacy of eHealth interventions among adults with DM, highlighting acceptable support in controlling HbA1c levels and thereby confirming previous studies [12,13]. Indeed, the evaluated interventions are mainly delivered using applications embedded in smartphones or tablets, designed to monitor and improve individuals’ metabolic control, physical activity, diabetes self-management, and the risk of hypoglycemia, as well as to reduce healthcare costs [31,32,33,34,35,36,37,38,39,40,41,42,43]. However, the beneficial effects of eHealth interventions on the metabolic control of people with DM was not maintained at follow-up. Several reasons can justify this loss of efficacy, first and foremost the lack of generalizability of the enhancements assigned to interventions, which could entail recidivism [44]. Indeed, generalizability is fundamental for the long-term effect of intervention since it assumes the transfer of behavioral change to other areas of an individual’s functioning. In addition, the difference between efficacy and effectiveness is also important to mention. Efficacy refers to the effect of intervention when assessed in a controlled condition, thus the preferred design for efficacy studies is the RCT. Indeed, RCTs carefully plan the conditions in which the intervention is tested and closely monitor and control any possible confounding factor, and then aim to generalize the results to the whole population [45]. Effectiveness, on the other hand, is tested in more ecological environments, meaning that the intervention’s effects are assessed imitating everyday conditions (e.g., not using randomized designs and not systematically excluding confounding variables); this allows for inter-contextual generalizability of the results [45].

As previously mentioned, the considered eHealth interventions were not developed to provide psychosocial support to improve adults’ well-being, and, indeed, no significant results concerning adults’ quality of life and diabetes distress have emerged, nor were data referred to anxiety and stress symptoms evaluated by any of the included RCTs. This is interesting considering that the American Association of Diabetes Educators (AADE) has provided guidelines for effective diabetes management, and thus to provide healthy behavioral strategies. More specifically, AADE refers to seven self-care behaviors [46]: healthy eating, being physically active, monitoring blood glucose levels, adhering to prescribed medications, adequate problem-solving skills, risk-reduction behaviors, and healthy coping. The latter is a construct that encompasses several interrelated psychosocial dimensions, namely diabetes distress, distress (subsuming depression, anxiety, and stress symptoms referred to life at large) and mental well-being (encompassing quality of life, positive attitudes, and positive relationships) [6]. These findings give rise to reflections on the importance of developing and delivering eHealth interventions suitable to provide psychosocial support to people with DM, using an RCT design to test their effectiveness; indeed, most research on eHealth interventions developed to provide psychosocial support consists of pilot or proof-of-concept studies [47,48,49].

Notwithstanding these shortcomings, results from this meta-analysis yielded encouraging evidence on the efficacy of eHealth interventions in decreasing depressive symptoms [31,32,36,37,42,43], albeit results show a small effect size. The literature suggests several possible biological mechanisms to explain the onset of depressive symptoms, which can then interfere with HbA1c levels. For instance, studies suggest that the effects of insulin deficiency on the metabolism of neurotransmitters may be at the basis of both depression and chronic high glycemic levels, which in turn have potentially hindering effects on the hypothalamic–pituitary–adrenal axis (HPA) [50,51]. The results of the present meta-analysis confirm and extend those previously emerged, suggesting a bidirectional relationship between depression and metabolic control, whereby eHealth interventions aimed at fostering glycemic control and improving HbA1c levels also lead to improvements in depressive symptoms. This bidirectional association exposes adults with DM to a group of various cardiovascular risk factors, comprising elevated blood pressure, hyperglycemia, obesity, hypertriglyceridemia, and decreased high density lipoprotein (HDL) cholesterol [52]. Indeed, the bidirectionality between psychosocial and medical factors is a pivotal aspect that should be taken into account to extend intervention efficacy to the whole functioning of an individual. A quite recent longitudinal study also suggested a “dynamic interaction” [53] (p. 952) between depressive symptoms and HbA1c levels, in which depressive symptoms may be risk factors for the increase of HbA1c levels and vice versa [53,54]. Moreover, depression can involve feelings of hopelessness and helplessness [55], which can influence the individual’s motivation to adopt healthy behaviors, namely following a healthy diet, doing regular physical activity, and taking oral medication and/or insulin injections. Adherence to the recommended regimen continues to represent a barrier for many people with DM: most of them, indeed, present difficulties in regularly engaging with all the aforementioned healthy behaviors, and this influences their diabetes management [56,57]. Therefore, not fully adhering to the prescribed medical regimens constitutes a risk factor from both a medical and psychosocial standpoint, since it can entail poor glycemic control in the short-term which, in turn, can have an impact on the psychosocial functioning of an individual.

Notwithstanding the differences in possible complications and management of T1DM and T2DM, it is noteworthy that the results of the studies included in the current meta-analysis highlighted no significant differences in eHealth interventions’ efficacy when compared considering the type of diabetes as moderator. These findings suggest that interventions might be focused on the broader concept of DM, concentrating on the similarities among patient’s lifestyles and making the self-management of metabolic control their main core. Indeed, only in one study was the intervention specifically focused on the risk of hypoglycemia among people with T1DM [34], thereby supporting this hypothesis.

The main limitation of the present meta-analysis is that most interventions were developed for monitoring and/or improving glycemic control in DM, except for four studies, in which the authors also designed an interactive diary to motivate the person to achieve a better quality of life [33,34] and to provide social support [31,32,33]. Therefore, the current meta-analysis could not assess the differences between eHealth interventions aimed at fostering psychosocial vs. medical support. Additionally, most studies currently available in the literature that have integrated the psychosocial factors within the digital health solutions are mostly pilot or proof-of-concept studies [47,48,49], thus not feasible for rigorous systematic reviews and meta-analyses. Therefore, future studies should move towards RCT designs to prove the effectiveness of eHealth interventions while also taking into account the bidirectionality between psychosocial and medical factors. Noteworthy is also the high risk of bias of the included evidence. Although the reduced RCTs quality was for the most caused by a lack in properly investigating adherence to intervention, all studies properly assessed the outcomes of interest and satisfactorily dealt with missing data. As such, the identified RCTs’ low quality mainly poses concern on the evaluation of interventions’ tolerability, albeit not greatly undermining the reliability of the results.

## 5. Conclusions

This meta-analysis provides a contribution to the growing literature on eHealth interventions for supporting and motivating people with DM in the adoption of healthy goals within the management of their chronic disease. Indeed, results highlighted that eHealth interventions aimed at monitoring individuals’ metabolic control and enhancing depressive symptoms are effective and acceptable in the short-term; however, no evidence on efficacy at follow-up was found. Future studies should design digital solutions following standard guidelines, such as those identified by AADE [46], thereby including the healthy coping construct within their intervention protocol. As such, future works should develop eHealth interventions on multiple levels, including a broad range of psychosocial factors to fully address the barriers of non-adherence as well as foster psychophysical well-being. Lastly, studies should develop new approaches to support the long-term maintenance of interventions’ efficacy related to glycemic levels as well as depressive symptoms.

## Figures and Tables

**Table 1:** Characteristics of the included studies.

			Sample Size			Intervention				
Author, Year	Country	Sample	Treatment	Control	Mean Age (Years); (SD)	Treatment	Control	Time Points	Outcomes Included	Data Type
[31]	USA	T2DM	97 CC	78	57.35 (10.60)	Telephone-based intervention	Active	Endpoint Follow-up (16 and 32 weeks)	HbA1c Diabetes distress Depression	ITT
[32]	USA	T2DM	93 IC	78	56.25 (10.91)	Telephone-based intervention	Active	Endpoint Follow-up (16 and 32 weeks)	HbA1c Diabetes distress Depression	ITT
[33]	Italy	T1DM	67	63	35.70 (9.40)	Mobile-based telemedicine system	TAU	Endpoint Follow-up (12 weeks)	HbA1c QoL	ITT
[34]	Italy	T1DM	63	64	36.90 (10.50)	Mobile-based telemedicine system	TAU	Endpoint	HbA1c QoL	ITT
[35]	France	T1DM	30	30	41.30 (11.30)	Mobile-phone software	Waitlist	Endpoint	HbA1c QoL	Per-protocol
[36]	USA	T2DM	25	25	60.00 (8.81)	Comprehensive telemedicine intervention	TAU	Endpoint Follow-up (13 weeks)	HbA1c Depression	ITT
[37]	Turkey	T2DM	23	21	52.93 (11.18)	Tele-rehabilitation	TAU	Endpoint	HbA1c Depression	Per-protocol
[38]	Spain	T1DM	54	64	31.85 (9.57)	Internet-based telemedicine system	TAU	Endpoint	HbA1c QoL	Per-protocol
[39]	USA	T2DM	93	95	51.50 (9.01)	Interactive web-based tablet, computer-delivered tool	Active	Endpoint	Diabetes distress HbA1c	Per-protocol
[40]	Spain	T1DM	19	16	25.00 (8.54)	Telephone-based telecare	TAU	Endpoint Follow-up (26 weeks)	HbA1c QoL	ITT
[41]	Australia	T1DM	36	36	35.20 (10.43)	Smartphone-based app	TAU	Endpoint Follow-up (12 weeks)	HbA1c QoL	ITT
[42]	USA	T2DM	62	35	54.09 (8.30)	Mobile Health intervention	Active	Endpoint	HbA1c Diabetes distress Depression QoL	Per-protocol
[43]	Australia	T2DM	63	63	61.30 (11.10)	Tailored telemonitoring intervention	TAU	Endpoint	HbA1c QoL Depression	Per-protocol

Note. USA = United States; CC = Couple Calls; IC = Individual Calls; T1DM = Type 1 Diabetes Mellitus; T2DM = Type 2 Diabetes Mellitus; TAU = Treatment as usual; HbA1c = Hemoglobin A1c; QoL = Quality of life; ITT = Intention-To-Treat.

**Table 2:** eHealth Interventions’ characteristics.

Author, Year	Treatment Length (Weeks)	Intervention	Type of Digital Intervention	Delivery Format	Aim
[31]	16	Telephonic couples or individual behavioural diabetes intervention	Telephone-based intervention	Phone calls	Improve glycaemic control, physical health, and psychological outcomes.
[32]	16	Telephonic couples or individual behavioural diabetes intervention	Telephone-based intervention	Phone calls	Improve glycaemic control, physical health, and psychological outcomes.
[33]	26	Diabetes Interactive Diary (DID) software	Mobile-based telemedicine system	Mobile phone software	Improve metabolic control, quality of life and reduces the risk of hypoglycaemia.
[34]	12	Diabetes Interactive Diary (DID) software	Mobile-based telemedicine system	Mobile phone software	Improve metabolic control while avoiding weight gain and reducing time devoted to education.
[35]	26	GlucoNet system	Mobile phone software	Short Message Service (SMS)	Improve metabolic control through telemonitoring.
[36]	26	Advanced Comprehensive Diabetes Care (ACDC) program	Comprehensive telemedicine intervention	Phone calls	Foster telemedicine-based management of clinic refractory Persistent Poorly Diabetes Mellitus (PPDM).
[37]	6	Tele-rehabilitation (TR) program	Tele-rehabilitation	Video calls	Improve glucose control, exercise capacity, physical fitness, muscle strength, and psychosocial status.
[38]	26	Medical Guard Diabetes (MGD) system	Internet-based telemedicine system	Web-based system	Improve metabolic control.
[39]	12	iDecide program	Web-based personally tailored, interactive diabetes medication decision aid	Tablet computer-delivered tool	Improve key diabetes outcomes by focusing on treatment barriers and diabetes management.
[40]	26	GlucoBeep device	Telephone-based telecare	Telephone device	Improve metabolic control and self-management.
[41]	26	Glucose Buddy app combined with weekly text-message feedback from a Certified Diabetes Educator (CDE)	Diabetes self-management iPhone application	Smartphone application	Improve glycaemic control and other diabetes-related outcomes.
[42]	26	Community-based diabetes self-management education (DSME) plus mobile health (mHealth)-enhanced peer support intervention	Mobile Health intervention	Web application	Foster changes in glycaemic control.
[43]	26	Townsville Broadband Diabetes Telehealth (TBDT) trial	Tailored telemonitoring intervention	Tablet computer-software	Improve glycaemic control and reduce healthcare costs.

**Table 3:** Sub-group and meta-regression analyses.

HbA1c					
Sub-Group Analysis					
	k	SMD	95% CI	I^2^	*p*
Control condition					
Waiting list	1	−0.25	−0.76, 0.26	-	0.10
TAU	8	−0.63	−1.08, −0.17	88%	
Active control	4	−0.08	−0.28, 0.11	31%	
Diabetes Type					
T1DM	6	−0.54	−1.16, 0.07	91%	0.08
T2DM	7	−0.27	−0.51, −0.03	63%	
Intervention delivery format					
SMS	1	−0.25	−0.76, 0.26	-	0.28
Phone calls	3	−0.25	−0.45, −0.05	0%	
Video calls	1	−0.88	−1.5, −0.26	-	
Applications	8	−0.42	−0.88, 0.04	91%	
Type of analyses					
ITT	7	−0.58	−1.04, −0.11	89%	0.18
Per-protocol	6	−0.20	−0.50, 0.10	85%	
**Meta-Regression**					
	**k**	**β**	**SE**	**z**	***p***
Age	13	0.02	0.02	0.97	0.33
Female gender	12	0.006	0.01	0.62	0.54
Diabetes duration	12	−0.02	0.04	−0.62	0.54
Intervention Duration	13	−0.007	0.03	−0.29	0.77

Note. HbA1c = Hemoglobin A1c; SMD = Standard Mean Difference; CI = Confidence Interval; I^2^ = Heterogeneity; TAU = Treatment as usual; T1DM = Type 1 Diabetes Mellitus; T2DM = Type 2 Diabetes Mellitus; SMS = Short Message Service; ITT = Intention-To-Treat; *p* < 0.05.

## Supplementary Materials

The following are available online at https://www.mdpi.com/article/10.3390/ijerph18178982/s1, Figure S1: PRISMA 2020 Checklist, Table S1: Excluded Studies with reasons, Figure S2: Risk of Bias Plot, Figure S3: Quality of Life at the endpoint, Figure S4: Diabetes Distress at the endpoint.
