# Supplementary material for: Efficacy of eHealth Interventions for Adults with Diabetes: A Systematic Review and Meta-Analysis
Source: Int J Environ Res Public Health. 2021 Aug 26;18(17):8982. doi: 10.3390/ijerph18178982 (PMC8431552; doi:10.3390/ijerph18178982)
Supplement: Supplementary file 1 [file ijerph-18-08982-s001.zip › ijerph-1329862-supplementary.pdf]

Supplementary Materials

Figure S1. PRISMA 2020 Checklist

Section and Topic	Item #	Checklist item	Location where item is reported
**TITLE**			
Title	1	Identify the report as a systematic review.	1
**ABSTRACT**			
Abstract	2	See the PRISMA 2020 for Abstracts checklist.	2
**INTRODUCTION**			
Rationale	3	Describe the rationale for the review in the context of existing knowledge.	3-5
Objectives	4	Provide an explicit statement of the objective(s) or question(s) the review addresses.	5
**METHODS**			
Eligibility criteria	5	Specify the inclusion and exclusion criteria for the review and how studies were grouped for the syntheses.	6
Information sources	6	Specify all databases, registers, websites, organisations, reference lists and other sources searched or consulted to identify studies. Specify the date when each source was last searched or consulted.	6
Search strategy	7	Present the full search strategies for all databases, registers and websites, including any filters and limits used.	6
Selection process	8	Specify the methods used to decide whether a study met the inclusion criteria of the review, including how many reviewers screened each record and each report retrieved, whether they worked independently, and if applicable, details of automation tools used in the process.	6-7
Data collection process	9	Specify the methods used to collect data from reports, including how many reviewers collected data from each report, whether they worked independently, any processes for obtaining or confirming data from study investigators, and if applicable, details of automation tools used in the process.	7
Data items	10a	List and define all outcomes for which data were sought. Specify whether all results that were compatible with each outcome domain in each study were sought (e.g. for all measures, time points, analyses), and if not, the methods used to decide which results to collect.	7
	10b	List and define all other variables for which data were sought (e.g. participant and intervention characteristics, funding sources). Describe any assumptions made about any missing or unclear information.	7
Study risk of bias assessment	11	Specify the methods used to assess risk of bias in the included studies, including details of the tool(s) used, how many reviewers assessed each study and whether they worked independently, and if applicable, details of automation tools used in the process.	7
Effect measures	12	Specify for each outcome the effect measure(s) (e.g. risk ratio, mean difference) used in the synthesis or presentation of results.	8
Synthesis methods	13a	Describe the processes used to decide which studies were eligible for each synthesis (e.g. tabulating the study intervention characteristics and comparing against the planned groups for each synthesis (item #5)).	8
	13b	Describe any methods required to prepare the data for presentation or synthesis, such as handling of missing summary statistics, or data conversions.	8
	13c	Describe any methods used to tabulate or visually display results of individual studies and syntheses.	8
	13d	Describe any methods used to synthesize results and provide a rationale for the choice(s). If meta-analysis was performed, describe the model(s), method(s) to identify the presence and extent of statistical heterogeneity, and software package(s) used.	8
	13e	Describe any methods used to explore possible causes of heterogeneity among study results (e.g. subgroup analysis, meta-regression).	8
	13f	Describe any sensitivity analyses conducted to assess robustness of the synthesized	NA

Section and Topic	Item #	Checklist item	Location where item is reported
		results.	
Reporting bias assessment	14	Describe any methods used to assess risk of bias due to missing results in a synthesis (arising from reporting biases).	8
Certainty assessment	15	Describe any methods used to assess certainty (or confidence) in the body of evidence for an outcome.	8
**RESULTS**			
Study selection	16a	Describe the results of the search and selection process, from the number of records identified in the search to the number of studies included in the review, ideally using a flow diagram.	8-9
	16b	Cite studies that might appear to meet the inclusion criteria, but which were excluded, and explain why they were excluded.	9 (Table 1_Supplementary Materials 1)
Study characteristics	17	Cite each included study and present its characteristics.	9-11
Risk of bias in studies	18	Present assessments of risk of bias for each included study.	12 & Figure 2
Results of individual studies	19	For all outcomes, present, for each study: (a) summary statistics for each group (where appropriate) and (b) an effect estimate and its precision (e.g. confidence/credible interval), ideally using structured tables or plots.	12-14
Results of syntheses	20a	For each synthesis, briefly summarise the characteristics and risk of bias among contributing studies.	12-14 & Figure 2
	20b	Present results of all statistical syntheses conducted. If meta-analysis was done, present for each the summary estimate and its precision (e.g. confidence/credible interval) and measures of statistical heterogeneity. If comparing groups, describe the direction of the effect.	12-14
	20c	Present results of all investigations of possible causes of heterogeneity among study results.	12-14
	20d	Present results of all sensitivity analyses conducted to assess the robustness of the synthesized results.	NA
Reporting biases	21	Present assessments of risk of bias due to missing results (arising from reporting biases) for each synthesis assessed.	12 & Figure 2
Certainty of evidence	22	Present assessments of certainty (or confidence) in the body of evidence for each outcome assessed.	14
**DISCUSSION**			
Discussion	23a	Provide a general interpretation of the results in the context of other evidence.	14-18
	23b	Discuss any limitations of the evidence included in the review.	17-18
	23c	Discuss any limitations of the review processes used.	17-18
	23d	Discuss implications of the results for practice, policy, and future research.	17-18
**OTHER INFORMATION**			
Registration and protocol	24a	Provide registration information for the review, including register name and registration number, or state that the review was not registered.	1
	24b	Indicate where the review protocol can be accessed, or state that a protocol was not prepared.	NA
	24c	Describe and explain any amendments to information provided at registration or in the protocol.	NA
Support	25	Describe sources of financial or non-financial support for the review, and the role of the funders or sponsors in the review.	1
Competing interests	26	Declare any competing interests of review authors.	1
Availability of data, code and other	27	Report which of the following are publicly available and where they can be found: template data collection forms; data extracted from included studies; data used for all analyses; analytic code; any other materials used in the review.	NA

Section and Topic	Item #	Checklist item	Location where item is reported
materials			

Table S1. Excluded Studies with reasons

N.	Author, year	Reason for exclusion
1	Agarwal, P., 2019	The study included participants with Diabetes Mellitus (DM) medical diseases.
2	Aikens, J.E., 2015	The study is not a Randomized Controlled Trial (RCT); the study's population did not meet the age range criterion of the current meta-analysis; participants with DM medical diseases (i.e., hypertension, cardiovascular disease, cancer, stroke, arthritis, chronic lung disease) were included.
3	Anderson, D.R., 2010	The study included participants with medical (i.e., hypertension and asthma) and psychological (i.e., depression) diseases.
4	Arora, S., 2013	The study included participants with medical (i.e., hearth or kidney disease, history of stroke, arthritis) and psychological (i.e., depression) diseases other than T1DM or T2DM.
5	Bailey, D.P., 2020	The study did not assess the pre-defined primary medical outcome (i.e., HbA1c) of the current meta-analysis.
6	Baron, J., 2015	This article only reported about preliminary data.
7	Baron, J., 2017	The study included participants with DM with a disease (i.e., "comorbidities", not otherwise specified).
8	Bertuzzi, F., 2018	The study's population did not meet the age range criterion of the current meta-analysis; the study assessed none of the pre-defined primary psychological outcomes of the current meta-analysis.
9	Boaz, M., 2009	The measure to assess anxiety, depression was not specified.
10	Boels, A.M., 2018	This article only reported about preliminary data.
11	Bonn, S.E., 2018	This article only reported about preliminary data.
12	Bujnowska-Fedak, M.M., 2011	The study's population did not meet the age range criterion of the current meta-analysis.
13	Cho, J.H., 2017	The study's population did not meet the age range criterion of the current meta-analysis.
14	Clark, T.L., 2020	The study's population did not meet the age range criterion of the current meta-analysis.

15	Döbler, A., 2018	The study's population did not meet the age range criterion of the current meta-analysis.
16	Dobson, R., 2018	The study's population did not meet the age range criterion of the current meta-analysis.
17	Doupis, J., 2018	This article's full text is not available online.
18	Drion, I., 2015	The authors used median and IQR with non-parametric analyses; therefore, following Cochrane handbook, it was not possible to turn into mean and standard deviation, respectively.
19	Egede, L.E., 2017a	The study's population did not meet the age range criterion of the current meta-analysis; the study assessed none of the pre-defined primary psychological outcomes of the current meta-analysis.
20	Egede, L.E., 2017b	The study's population did not meet the age range criterion of the current meta-analysis; the study assessed none of the pre-defined primary psychological outcomes of the current meta-analysis.
21	Egede, L.E., 2018	The study's population did not meet the age range criterion of the current meta-analysis; inclusion of participants with DM with a psychological disease (i.e., depressive disorder).
22	Fortmann, A.L., 2016	This article is a conference abstract.
23	Gong, E., 2020	The study included participants with DM with a medical disease (i.e., "diagnosed comorbidities" not otherwise specified).
24	Heitkemper, E., 2017	This article's full text is not available online.
25	Hilmarsdóttir, 2020a	The study's population did not meet the age range criterion of the current meta-analysis.
26	Hilmarsdóttir, 2020b	This article is a conference abstract.
27	Holland-Carter, L., 2017	The study's population did not meet the age range criterion of the current meta-analysis.
28	Holmen, H., 2014	The study included participants with DM with medical diseases (i.e., "comorbidities", not otherwise specified).
29	Holmen, H., 2015	This article is a conference abstract.
30	Holmen H., 2016	The study's population did not meet the age range criterion of the current meta-analysis; inclusion of participants with DM with a disease (i.e., "comorbidities", not otherwise specified).
31	Izquierdo, R.E., 2003	The study's population did not meet the age range criterion of the current meta-analysis.
32	Kardas, P., 2016	The study included participants with DM with medical diseases (i.e., hypertension).
33	Kaur R., 2015	The study's design (e.g., RCT) is not clearly stated and it does not include an eHealth intervention.

34	Kempf, K., 2017	The study's population did not meet the age range criterion of the current meta-analysis.
35	Kumar, D., 2018	The study assessed none of the pre-defined primary medical nor psychological outcomes of the current meta-analysis.
36	Kumar, D.S., 2020	The study did not assess the pre-defined primary medical outcome of the current meta-analysis.
37	Logan, A.G., 2012	The study included participants with DM with a medical disease (i.e., hypertension and cardiovascular disease) and did not assess the pre-defined primary medical outcome of the current meta-analysis.
38	Mayberry, L.S., 2019	The study is not a RCT and does not include an eHealth intervention.
39	Mora, P., 2017	The study is not a RCT.
40	Nicolucci, A., 2015	The study included participants with DM with a medical disease (i.e., hypertension, dyslipidemia and cardiovascular complications).
41	Nobis, S., 2015	The study's population did not meet the age range criterion of the current meta-analysis; inclusion of participants with DM with a psychological disease (i.e., depression); the study did not assess the pre-defined primary medical outcome of the current meta-analysis.
42	Noviani, L., 2020	This article is a conference abstract (not available online).
43	Peiris, D., 2016	This article only reported about preliminary data.
44	Piette, J.D., 2000	The study did not assess the pre-defined primary medical outcome of the current meta-analysis; the study did not assess the pre-defined primary medical outcome of the current meta-analysis.
45	Polonsky, W.H., 2020	This study is not a RCT.
46	Poppe, L., 2019	The study assessed none of the pre-defined primary medical nor psychological outcomes of the current meta-analysis.
47	Quinn, C.C., 2011	The study included participants with DM with a medical disease (i.e., hypertension and coronary artery disease).
48	Quinn, C.C., 2017	The study included participants with DM with a medical disease (i.e., hypertension and coronary artery disease).
49	Ramallo-Fariña, Y., 2015	This article only reported about preliminary data.

50	Skrøvseth, S.O., 2015	The study assessed none of the pre-defined primary psychological outcomes of the current meta-analysis.
51	Tang, P.C., 2013	The study's population did not meet the age range criterion of the current meta-analysis.
52	Torbjørnsen, A., 2014	The study included participants with DM with a disease (i.e., "comorbidities", not otherwise specified).
53	Torbjørnsen, 2015	This article is a conference abstract.
54	Trief, P.M., 2006	The study's population did not meet the age range criterion of the current meta-analysis.
55	Van Bastelaar, K.M.P., 2011	The study included participants with DM with a psychological disease (i.e., depression).
56	Van der Weegen, S., 2015	The study's population did not meet the age range criterion of the current meta-analysis; inclusion of participants with DM with a medical disease (i.e., chronic obstructive pulmonary disease).
57	Von Storch, K., 2019	The study's population did not meet the age range criterion of the current meta-analysis; inclusion of participants with DM with a medical disease (i.e., "multimorbidities", specified as "chronic diseases"); the study assessed none of the pre-defined psychological outcomes of the current meta-analysis.
58	Wang, Y., 2019	The study assessed none of the pre-defined primary medical nor psychological outcomes of the current meta-analysis.
59	Wayne, N., 2015	The study's population did not meet the age range criterion of the current meta-analysis; inclusion of participants with DM with psychiatric diagnoses.
60	Weinstock, R.S., 2011	The study's population did not meet the age range criterion of the current meta-analysis; inclusion of participants with a disease other than T1 or 2 Diabetes (i.e., "comorbidities", not otherwise specified).
61	Whittemore, R., 2019	This article is a conference abstract.
62	Williams, E.D., 2012	The study's population did not meet the age range criterion of the current meta-analysis; inclusion of participants with DM with a medical disease (i.e., hypertension, kidney disease, cardiovascular disease).

63	Yaron, M., 2019	The study included participants with DM with a medical disease (i.e., hypertension, hyperlipidemia).
64	Zhang, L., 2018	This article is a conference abstract.

1. Agarwal, P. (2019). Mobile App for Improved Self-Management of Type 2 Diabetes: Multicenter Pragmatic Randomized Controlled Trial. doi: 10.2196/10321
2. Aikens, J.E. (2015). Improvements in illness self-management and psychological distress associated with telemonitoring support for adults with diabetes. doi: 10.1016/j.pcd.2014.06.003
3. Anderson, D.R. (2010). Managing the Space between Visits: A Randomized Trial of Disease Management for Diabetes in a Community Health Center. doi: 10.1007/s11606-010-1419-5
4. Arora, S. (2013). Trial to Examine Text Message–Based mHealth in Emergency Department Patients With Diabetes (TExT-MED): A Randomized Controlled Trial. doi: 10.1016/j.annemergmed.2013.10.012
5. Bailey, D.P. (2020). Randomised Controlled Feasibility Study of the MyHealthAvatar-Diabetes Smartphone App for Reducing Prolonged Sitting Time in Type 2 Diabetes Mellitus. doi: 10.3390/ijerph17124414
6. Baron, J. (2015). A Mobile Telehealth Intervention for Adults With Insulin-Requiring Diabetes: Early Results of a Mixed-Methods Randomized Controlled Trial. doi: 10.2196/resprot.4035
7. Baron, J. (2017). A randomised, controlled trial of the effects of a mobile telehealth intervention on clinical and patient-reported outcomes in people with poorly controlled diabetes. doi: 10.1177/1357633X16631628
8. Bertuzzi, F. (2018). Teleconsultation in type 1 diabetes mellitus (TELEDIABE). doi: 10.1007/s00592-017-1084-9
9. Boaz, M. (2009). An Automated Telemedicine System Improves Patient-Reported Well-Being. doi: 10.1089/dia.2008.0048
10. Boels, A.M. (2018). Effectiveness of diabetes self-management education via a smartphone application in insulin treated type 2 diabetes patients – design of a randomised controlled trial ('TRIGGER study'). doi: 10.1186/s12902-018-0304-9
11. Bonn, S.E. (2018). App-technology to increase physical activity among patients with diabetes type 2 - the DiaCert-study, a randomized controlled trial. doi: 10.1186/s12889-018-5026-4
12. Bujnowska-Fedak, M.M. (2011). The Impact of Telehome Care on Health Status and Quality of Life Among Patients with Diabetes in a Primary Care Setting in Poland. doi: 10.1089/tmj.2010.0113
13. Cho, J.H., (2017). An Internet-based health gateway device for interactive communication and automatic data uploading: Clinical efficacy for type 2 diabetes in a multi-centre trial. doi: 10.1177/1357633X16657500
14. Clark, T.L. (2020). Does Diabetes Distress Influence Clinical Response to an mHealth Diabetes Self-Management Education and Support Intervention?. doi: 10.1177/0145721720913276
15. Döbler, A. (2018). Telephone-Delivered Lifestyle Support With Action Planning and Motivational Interviewing Techniques to Improve Rehabilitation Outcomes. doi: 10.1037/rep0000224
16. Dobson, R. (2018). Effectiveness of text message based, diabetes self management support programme (SMS4BG): two arm, parallel randomised controlled trial. doi: 10.1136/bmj.k1959
17. Doupis, J. (2018). Mobile-based artificial intelligence significantly improves type 1 diabetes management. doi: 10.2337/db18-1058-P
18. Drion, I. (2015). The Effects of a Mobile Phone Application on Quality of Life in Patients With Type 1 Diabetes Mellitus: A Randomized Controlled Trial. doi: 10.1177/1932296815585871
19. Egede, L.E. (2017a). Telephone-Delivered Behavioral Skills Intervention for African American Adults with Type 2 Diabetes: A Randomized Controlled Trial. doi: 10.1007/s11606-017-4023-0
20. Egede, L.E. (2017b). Randomized Controlled Trial of Technology-Assisted Case Management in Low Income Adults with Type 2 Diabetes. doi: 10.1089/dia.2017.0006
21. Egede, L.E. (2018). Effect of psychotherapy for depression via home telehealth on glycemic control in adults with type 2 diabetes: Subgroup analysis of a randomized clinical trial. doi: 10.1177/1357633X17730419
22. Fortmann, A.L. (2016). Diabetes distress affects responsiveness to an mhealth self-management intervention among hispanics with type 2 diabetes (dulce digital). doi: 10.2337/db16-652-860

23. Gong, E. (2020). My Diabetes Coach, a Mobile App–Based Interactive Conversational Agent to Support Type 2 Diabetes Self-Management: Randomized Effectiveness-Implementation Trial. doi: 10.2196/20322
24. Heitkemper, E. (2017). Characteristics of underserved adults enrolled in the mobile diabetes detective (MoDD) randomized controlled trial. doi: N.A.
25. Hilmarsdóttir, E. (2020a). A Digital Lifestyle Program in Outpatient Treatment of Type 2 Diabetes: A Randomized Controlled Study. doi: 10.1177/1932296820942286
26. Hilmarsdóttir, E. (2020b). A digital lifestyle program to support outpatient treatment of type 2 diabetes: a randomized controlled trial. doi: 10.1177/1932296819897652
27. Holland-Carter, L. (2017). Impact on psychosocial outcomes of a nationally available weight management program tailored for individuals with type 2 diabetes: Results of a randomized controlled trial. doi: 10.1016/j.jdiacomp.2017.01.022
28. Holmen, H. (2014). A Mobile Health Intervention for Self-Management and Lifestyle Change for Persons With Type 2 Diabetes, Part 2: One-Year Results From the Norwegian Randomized Controlled Trial RENEWING HEALTH. doi: 10.2196/mhe.alth.3882
29. Holmen, H. (2015). Patient-reported outcomes and the use of a diabetes diary mobile application to attain lifestyle changes for persons with type 2 diabetes. doi: 10.1007/s11136-015-1078-4
30. Holmen, H. (2016). Stages of change for physical activity and dietary habits in persons with type 2 diabetes included in a mobile health intervention: the Norwegian study in RENEWING HEALTH. doi: 10.1136/bmjdrc-2016-000193
31. Izquierdo, R.E. (2003). A Comparison of Diabetes Education Administered Through Telemedicine Versus in Person. doi: 10.2337/diacare.26.4.1002
32. Kardas, P. (2016). Type 2 Diabetes Patients Benefit from the COMODITY12 mHealth System: Results of a Randomised Trial. doi: 10.1007/s10916-016-0619-x
33. Kaur, R. (2015). Telephonic Consultation and follow-up in Diabetics: Impact on Metabolic Profile, Quality of Life, and Patient Compliance. doi: 10.4103/1947-2714.157483
34. Kempf, K. (2017). Efficacy of the Telemedical Lifestyle intervention Program TeLiPro in Advanced Stages of Type 2 Diabetes: A Randomized Controlled Trial. doi: 10.2337/dc17-0303
35. Kumar, D. (2018). Effectiveness of Randomized Control Trial of Mobile Phone Messages on Control of Fasting Blood Glucose in Patients with Type-2 Diabetes Mellitus in a Northern State of India. doi: 10.4103/ijph.IJPH_199_17
36. Kumar, D.S. (2020). An android smartphone-based randomized intervention improves the quality of life in patients with type 2 diabetes in Mysore, Karnataka, India. doi: 10.1016/j.dsx.2020.07.025
37. Logan, A.G. (2012). Effect of Home Blood Pressure Telemonitoring With Self-Care Support on Uncontrolled Systolic Hypertension in Diabetics. doi: 10.1161/HYPERTENSIONAHA.111.188409
38. Mayberry, L.S. (2019). Out-of-home informal support important for medication adherence, diabetes distress, hemoglobin A1c among adults with type 2 diabetes. doi: 10.1007/s10865-018-0002-0
39. Mora, P. (2017). Use of a Novel, Remotely Connected Diabetes Management System Is Associated with Increased Treatment Satisfaction, Reduced Diabetes Distress, and Improved Glycemic Control in Individuals with Insulin-Treated Diabetes: First Results from the Personal Diabetes Management Study. doi: 10.1089/dia.2017.0206
40. Nicolucci, A. (2015). A Randomized Trial on Home Telemonitoring for the Management of Metabolic and Cardiovascular Risk in Patients with Type 2 Diabetes. doi: 10.1089/dia.2014.0355
41. Nobis, S. (2015). Efficacy of a Web-Based Intervention With Mobile Phone Support in Treating Depressive Symptoms in Adults With Type 1 and Type 2 Diabetes: A Randomized Controlled Trial. doi: 10.2337/dc14-1728

42. Noviani, L. (2020). Collaboration between interprofessional healthcare and patients to improve quality of life type 2 diabetes via smartphone application. doi: 10.31838/ijpr/2021.13.01.126
43. Peiris, D. (2016). Systematic medical assessment, referral and treatment for diabetes care in China using lay family health promoters: protocol for the SMARTDiabetes cluster randomised controlled trial. doi: 10.1186/s13012-016-0481-8
44. Piette, J.D. (2000). The Effect of Automated Calls With Telephone Nurse Follow-Up on Patient-Centered Outcomes of Diabetes Care. A Randomized, Controlled Trial. doi: 10.1097/00005650-200002000-00011
45. Polonsky, W.H. (2020). Impact of Real-Time Continuous Glucose Monitoring Data Sharing on Quality of Life and Health Outcomes in Adults with Type 1 Diabetes. doi: 10.1089/dia.2020.0466
46. Poppe, L. (2019). Efficacy of a Self-Regulation–Based Electronic and Mobile Health Intervention Targeting an Active Lifestyle in Adults Having Type 2 Diabetes and in Adults Aged 50 Years or Older: Two Randomized Controlled Trials. doi: 10.2196/13363
47. Quinn, C.C. (2011). Cluster-Randomized Trial of a Mobile Phone Personalized Behavioral Intervention for Blood Glucose Control. doi: 10.2337/dc11-0366
48. Quinn, C.C. (2017). The Impact of a Mobile Diabetes Health Intervention on Diabetes Distress and Depression Among Adults: Secondary Analysis of a Cluster Randomized Controlled Trial. doi: 10.2196/mhealth.8910
49. Ramallo-Fariña, Y. (2015). Effectiveness and cost-effectiveness of knowledge transfer and behavior modification interventions in type 2 diabetes mellitus patients—the INDICA study: a cluster randomized controlled trial. doi: 10.1186/s13012-015-0233-1
50. Skrøvseth, S.O. (2015). Data-Driven Personalized Feedback to Patients with Type 1 Diabetes: A Randomized Trial. doi: 10.1089/dia.2014.0276
51. Tang, P.C. (2013). Online disease management of diabetes: Engaging and Motivating Patients Online With Enhanced Resources-Diabetes (EMPOWER-D), a randomized controlled trial. doi: 10.1136/amiajnl-2012-001263
52. Torbjørnsen, A. (2014). A Low-Intensity Mobile Health Intervention With and Without Health Counseling for Persons With Type 2 Diabetes, Part 1: Baseline and Short-Term Results From a Randomized Controlled Trial in the Norwegian Part of RENEWING HEALTH. doi: 10.2196/mhealth.3535
53. Torbjørnsen, A. (2015). Patient reported outcomes (PRO) of acceptability with the use of a diabetes mobile application. doi: 10.1007/s11136-015-1078-4
54. Trief, P.M. (2006). Depression and Glycemic Control in Elderly Ethnically Diverse Patients With Diabetes. doi: 10.2337/diacare.29.04.06.dc05-1769
55. Van Bastelaar, K.M.P. (2011). Web-Based Depression Treatment for Type 1 and Type 2 Diabetic Patients. doi: 10.2337/dc10-1248
56. Van der Weegen, S. (2015). It's LiFe! Mobile and Web-Based Monitoring and Feedback Tool Embedded in Primary Care Increases Physical Activity: A Cluster Randomized Controlled Trial. doi: 10.2196/jmir.4579
57. Von Storch, K. (2019). Telemedicine-Assisted Self-Management Program for Type 2 Diabetes Patients. DOI: 10.1089/dia.2019.0056
58. Wang, Y. (2019). Effects of continuous care for patients with type 2 diabetes using mobile health application: A randomised controlled trial. doi: 10.1002/hpm.2872
59. Wayne, N. (2015). Health Coaching Reduces HbA1c in Type 2 Diabetic Patients From a Lower-Socioeconomic Status Community: A Randomized Controlled Trial. doi: 10.2196/jmir.4871
60. Weinstock, R.S. (2011). Lessened decline in physical activity and impairment of older adults with diabetes with telemedicine and pedometer use: results from the IDEATel study. doi: 10.1093/ageing/afq147
61. Whittemore, R. (2019). Yo puedo! A self-management group and mHealth program for low-income adults with type 2 diabetes in Mexico City. doi: 10.2337/db19-339-OR

62. Williams, E.D. (2012). Randomised controlled trial of an automated, interactive telephone intervention (TLC Diabetes) to improve type 2 diabetes management: baseline findings and six-month outcomes. doi: 10.1186/1471-2458-12-602
63. Yaron, M. (2019). A randomized controlled trial comparing a telemedicine therapeutic intervention with routine care in adults with type 1 diabetes mellitus treated by insulin pumps. doi: 10.1007/s00592-019-01300-1
64. Zhang, L. (2018). A randomized controlled trial of a smart phone-based diabetes management application to improve blood glucose control in Chinese people with diabetes. doi: 10.1111/jdi.12939

Figure S2. Risk of Bias Plot

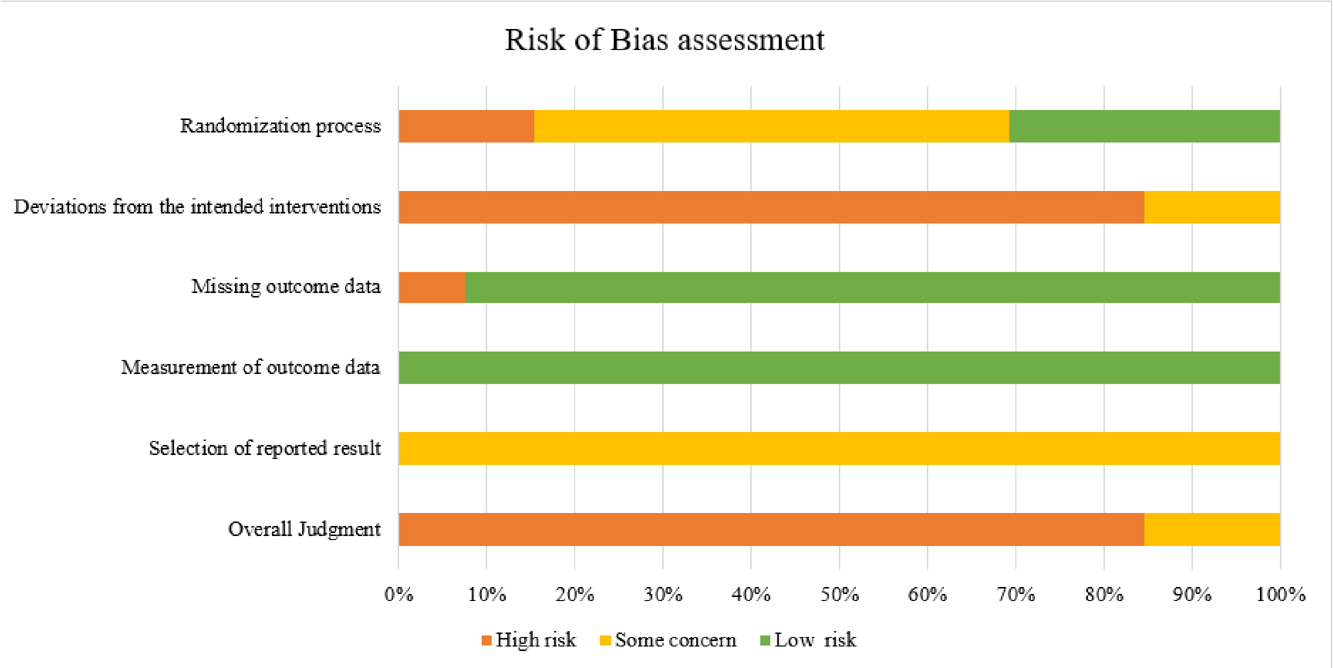

Figure S3. Quality of Life at the endpoint

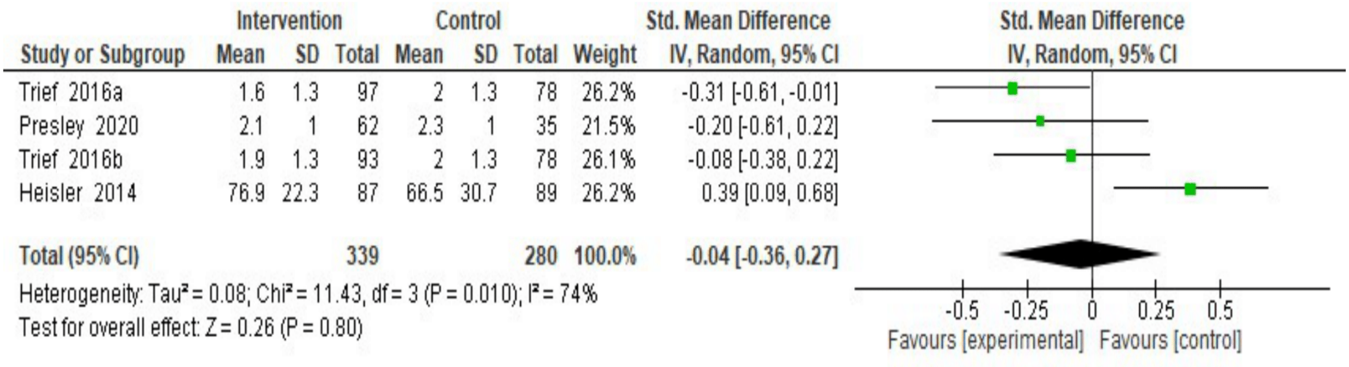

Note. SD = Standard Deviation; Std. Mean Difference = Standard Mean Difference; CI = Confidence Interval; df = degrees of freedom.

Figure S4. Diabetes Distress at the endpoint

Study or Subgroup	Intervention Mean	Intervention SD	Intervention Total	Control Mean	Control SD	Control Total	Weight	Std. Mean Difference IV, Random, 95% CI
Rossi 2013	69.1	2.2	63	71.7	0.09	63	14.8%	-1.66 [-2.07, -1.25]
Jansà 2006	37	3	19	37	4	16	12.8%	0.00 [-0.67, 0.67]
Warren 2018	0.65	0.08	63	0.65	0.09	63	15.1%	0.00 [-0.35, 0.35]
Presley 2020	63.4	22.9	62	62.6	17	35	14.7%	0.04 [-0.38, 0.45]
Benhamou 2007	69.3	9.4	17	68.8	9.3	22	13.1%	0.05 [-0.58, 0.69]
Rossi 2010	48.65	7.91	30	48.35	2.2	64	14.6%	0.06 [-0.37, 0.50]
Esmatjes 2014	69.9	18.7	50	66.9	17.4	50	14.9%	0.16 [-0.23, 0.56]
**Total (95% CI)**			**304**			**313**	**100.0%**	**-0.20 [-0.72, 0.32]**

Heterogeneity: Tau² = 0.44; Chi² = 57.63, df = 6 (P < 0.00001); I² = 90%
Test for overall effect: Z = 0.75 (P = 0.46)

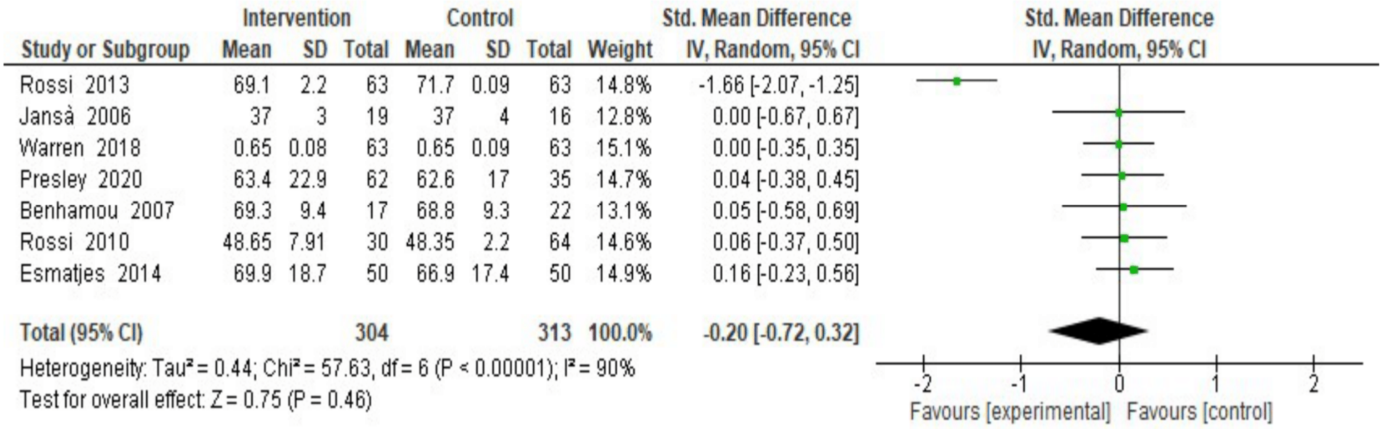

Note. SD = Standard Deviation; Std. Mean Difference = Standard Mean Difference; CI = Confidence Interval; df = degrees of freedom.
